# Federated Learning for Medical Image Analysis with Deep Neural Networks

**DOI:** 10.3390/diagnostics13091532

**Published:** 2023-04-24

**Authors:** Sajid Nazir, Mohammad Kaleem

**Affiliations:** 1 Department of Computing, Glasgow Caledonian University, Glasgow G4 0BA, UK; 2 Department of Electrical and Computer Engineering, COMSATS University Islamabad, Islamabad 45550, Pakistan

**Keywords:** deep neural networks, disease diagnosis, data privacy, model generalization, cryptography, blockchain

## Abstract

Medical image analysis using deep neural networks (DNN) has demonstrated state-of-the-art performance in image classification and segmentation tasks, aiding disease diagnosis. The accuracy of the DNN is largely governed by the quality and quantity of the data used to train the model. However, for the medical images, the critical security and privacy concerns regarding sharing of local medical data across medical establishments precludes exploiting the full DNN potential for clinical diagnosis. The federated learning (FL) approach enables the use of local model’s parameters to train a global model, while ensuring data privacy and security. In this paper, we review the federated learning applications in medical image analysis with DNNs, highlight the security concerns, cover some efforts to improve FL model performance, and describe the challenges and future research directions.

## 1. Introduction

Medical image modalities, such as ultrasound, X-rays, Magnetic Resonance Imaging (MRI), play a crucial role in disease diagnosis, and are used for diagnosing human body ailments, diseases, and various forms of cancers [1,2]. Increasingly, the disease diagnosis using the medical images is based on trained deep learning models. Deep Neural Networks (DNN) provide a state-of-the-art performance in medical image classification and segmentation tasks [2]. The model’s generalization performance is improved with diverse, and large-scale data [3]. The lack of sufficient data can be addressed by data augmentation [4], extracting the salient features from a small dataset [5], and use of Generative Adversarial Networks (GANs) [6].

The European General Data Protection Regulation (GDPR) [7] and the United States Health Insurance Portability and Accountability Act (HIPAA) [8] regulate the use and sharing of personal health information. The multi-national and multi-institutional data sharing for training a centralized model is limited due to the regulatory constraints. Therefore, unlike the data that can be publicly shared, trained, and analyzed by collecting it centrally, privacy constraints prohibit medical data sharing, and therefore the DNN models are constrained to the local data only. The proprietary nature and importance of the image datasets for medical diagnosis research limits the sharing of these with competitors or other institutions [9]. Even if the privacy and regulatory matters were addressed using encryption, the data migration to a central storage is not simple, as the image datasets are often very large [10,11].

**Figure 1:**
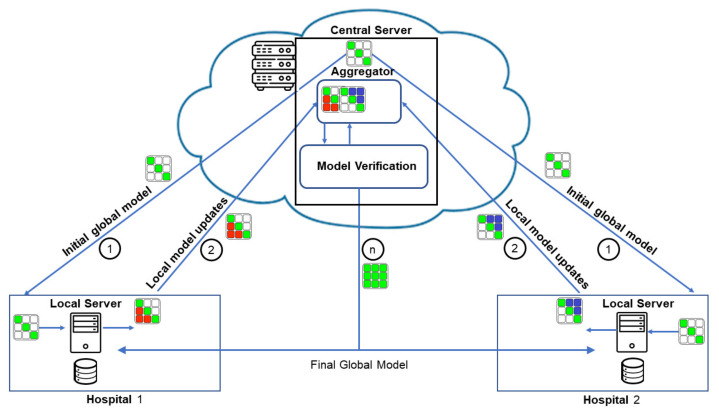
The general federated learning process. There would generally be more than two participating clients and the training rounds (shown as step 1 and 2). The model verification at the central and local participating clients takes place in each round.

Federated learning (FL) is a decentralized and collaborative approach, which does not need the local data to be shared. Instead, it is used to train a model locally, with only the model parameters shared with a central server as shown in Figure 1. The concept of FL was introduced by Google in 2017 and FedAvg algorithm was proposed for training the central server with the mobile phone data [12]. The FedAvg algorithm, unlike a centralized DNN model, made it possible to train the model without sharing the data [12]. The FL principles, however, can be applied for millions of devices termed as a cross-device scenario, or with fewer large establishments with relatively larger datasets, termed as a cross-silo scenario.

The benefit of FL is that it provides comparable results to centralized approaches, while ensuring data privacy. In a study for COVID-19 detection with Chest X-ray (CXR) images, non-independent and identically distributed (IID) and unbalanced data distributions were investigated with VGG-16 and ResNet50 models, and showed that the proposed framework was resilient and provided comparable performance to the centralized approaches [9].

FL has many applications in healthcare, such as for Electronic Health Records (EHR) [13], Internet of Medical Things (IoMT) [14], wearable healthcare [15], and medical imaging [13,16,17]. The FL approach requires the model training to be decentralized and collaborative, and can also work with videos [18]. Although the training data itself is not shared with the central server, and instead the model parameters of the locally trained model are communicated to it; however, encryption can be used as well to provide protection against eavesdropping on the model updates. A useful guide for designing and implementing FL optimization algorithms recommended a co-design of optimization with security and privacy issues [19].

The focus of this paper is to survey the use of FL approaches for medical image analysis with DNNs. We cover the state-of-the-art recent developments in this growing research field. In comparison to other survey papers, the major contributions of this paper are:

- Coverage of FL techniques for medical image segmentation and classification with DNNs for disease diagnosis with various image modalities;
- An overview of the security and performance and how these can be addressed;
- A discussion of the FL challenges and research directions for the FL application for medical imaging.

Rest of the paper is organized as follows: The background on FL is provided in Section 2. Section 3 provides a detailed coverage of the use of FL for medical imaging. The research challenges in the field are described in Section 4, and finally Section 5 concludes the paper.

## 2. Federated Learning

The FL breaks down the shortcoming of isolated data silos as the data can potentially be located anywhere in the world and yet be used for the global model learning, hence providing privacy preservation of the local data.

It would seem reasonable to train each of the local models on the local data and aggregate the models’ parameters to create a global model; however, in practice this would lead to poor performance across all data [16]. The FL model training of the local and global models is iteratively executed for many rounds and terminates based on the achievement of a performance threshold.

The initial model is obtained from the central server. This model is then trained on the local data of each of the participating local clients. After the local model has been trained, the model gradients are shared with the central server. The central server will wait for the gradients from the other participating clients. These gradients are then used by an algorithm such as FedAvg [12] to aggregate the global model. The aggregated global model is then shared with the participating clients, completing one round of the learning process. This process is repeated until the global model accuracy achieves the required threshold. The participating clients and the central server maintain the models from the previous iterations, and this could be a factor in deciding whether to update to the new model or instead use a previous version of the model.

Similarly, there are multiple considerations to initiate the model training process and the role of the central server. The model aggregation can also wait until a certain number of clients have contributed to the model updates.

The DNN model’s performance on the training or seen data is generally not important, whereas we are interested in the trained model’s ability to generalize to the unseen data. With more and diverse data available in FL, the biases due to demographics, type of equipment, etc., can be reduced and better model generalization can be obtained. An FL model for breast density classification was shown to provide 45.8% relative improvement in generalizability [17].

## 3. Federated Learning in Medical Imaging

**Figure 2:**
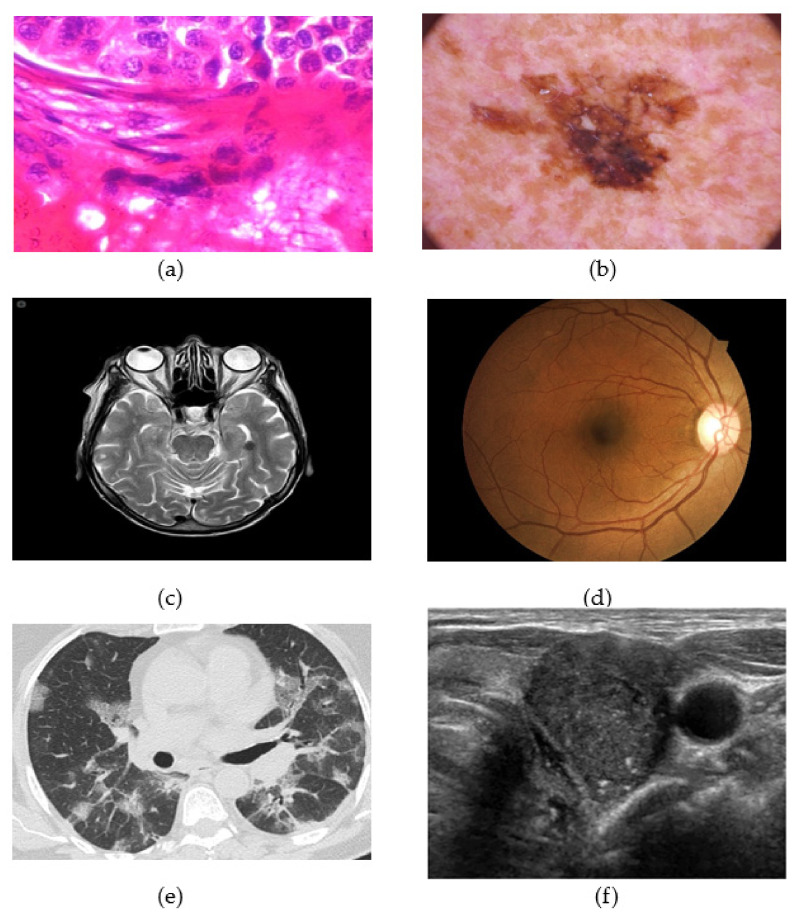
(**a**) Histopathological image showing malignant ductal carcinoma for Breast Cancer, (**b**) Skin Cancer, (**c**) MRI scans showing brain tumor, (**d**) Diabetic Retinopathy, (**e**) CT image for COVID-19 detection, (**f**) Thyroid ultrasound.

This section provides a survey of the recent research literature on the use of FL techniques applied to medical imaging, addressing security challenges associated with FL, and highlighting the performance improvements to the FL process. Some sample images are provided in Figure 2 which shows the range of image modalities employed for medical image diagnosis, indicative of the rich feature details required to be delineated for diagnosis.

### 3.1. FL Applications for Segmentation and Classification of Various Diseases

The literature on addressing various diseases are categorized in this sub-section. Model accuracy is often reported as a performance metric and we have included this in the following sections as reported by the different studies for their proposed techniques.

#### 3.1.1. COVID-19

Respiratory diseases such as COVID-19 and tuberculosis are commonly diagnosed using CXR and Computed Tomography (CT) images. Compared to CT, CXR images are easier to obtain using portable machines which are widely available. The application of FL for diagnosing respiratory diseases are described below with a summary provided in Table 1.

A dynamic focus-based FL framework FedFocus was proposed for COVID-19 detection with CXR images [20]. The focus of the study was to improve the model’s stability and accuracy. The training loss of each local model was considered for parameter aggregation [20]. It was shown that the proposed scheme outperformed the baseline methods and also achieved a faster convergence rate compared to FedAvg [20]. Another study used a dynamic fusion-based architecture for COVID-19 detection that was aimed at improving the communications efficiency and model performance in the presence of data heterogeneity [21]. The study used CXR and CT images and showed that the proposed method achieved better performance in accuracy and training time compared to the default FL [21]. FL was used for COVID-19 detection with CT images using a multinational study to investigate the model generalizability to the unseen data [22]. The best generalization performance achieved 95.66% accuracy with a FL model [22]. In another study, capsule network-based model, SegCaps was used for segmentation and classification of CT images for COVID-19 detection, showing better classification results compared to centralized methods with six DNN models, such as, VGG16, and DenseNet [23]. The study proposed data normalization for overcoming data heterogeneity and blockchain for data authentication [23]. A FL Ensembled Deep Learning Blockchain (FLED-Block) model was proposed comprising of an ensemble of VGG-16 and 19, Alexnets, Resnets-50 and 100, Resnets-50 and 100, Inception V3, Densenets-121, 119, and 150, Mobilenets, with SegCaps achieving an accuracy of 98.2% for COVID-19 prediction [24]. The study used capsule networks for feature extraction, and extreme learning machines (ELM) for classification, and blockchain for secure data retrieval [24].

CXR images were used for COVID-19 detection by implementing FL on Raspberry Pi 4 devices to investigate the use of low-power edge devices for lung segmentation [14]. The lung segmentation results on Raspberry Pi 4 devices were better for lung segmentation compared to a centralized approach [14]. IoMT-based framework was proposed for COVID-19 detection using the Flower dataset [25]. The Xception model achieved a global accuracy of 99.59% with three rounds [25]. An edge cloud-based solution, FedGAN for COVID-19 detection was proposed with Generative Adversarial Networks (GAN) to simulate the COVID-19 data distribution. The results showed better detection performance compared to the state-of-the-art solutions, that was attributable to the combination of GAN with FL [6]. A FL framework was proposed for COVID-19 classification on CXR images with pre-trained VGG16 and ResNet50 models [9]. COVID-19 detection with Non-IID and unbalanced data distributions [9]. The FL model performance was comparable to the centralized approach and was shown to increase with data augmentation [9]. A CXR dataset COVID-FL was created for COVID-19 classification, with a transformer-based self-supervised learning model [4]. The proposed method achieved an improvement of 4.58% in test accuracy on strongly skewed data distribution, in comparison to the supervised baselines [4].

A model, FedSGDCOVID, was proposed using FedAvg algorithm via local stochastic gradient descent (SGD) for COVID-19 detection [26]. SDG is a scalable optimization method and was used with differential privacy for controlling the effect of training data during the training for the large dataset used [26]. The proposed method had better performance compared to the other selected models, with an accuracy of 95.32% on CXR data. For CXR dataset, the model accuracy increased by 18.41% for the non-IID data [26].

An open-source framework, Privacy preserving Medical Imaging Analysis (PriMIA) was proposed for privacy-preserving FL [27]. The framework was evaluated theoretically and experimentally for classification of pediatric pneumonia using CXR images demonstrating similar classification performance to non-secure FL [27]. The PriMIA framework also provided privacy guarantees against gradient attacks [27]. An investigation of different number of clients and intermittent clients for pneumonia classification of CXR images used a multilayer CNN model showing increased security and reduced computation time compared to a centralized approach [28]. Federated Partially Supervised Learning (FPSL) was used as the basis of the proposed FedPSL framework to overcome the issue of limited data for FL [29]. The study utilized three public CXR datasets for COVID-19, Tuberculosis, and chest disease detection, and the evaluation of the effects of data scarcity, and clients with significantly different dataset sizes, showed better performance compared to FedAvg, and other selected techniques [29].

**Table 1:** FL applications for respiratory diseases.

Disease	Dataset/Model	Study Focus	Ref
COVID-19	Radiography Database, JSRT	COVID-19 detection on low-end devices	[14]
	CXR dataset and Symptoms dataset/four models including ResNet18 and ResNet50	Effect of IID and non-IID distributions	[26]
	3 lung CT image datasets/Capsule ensembled Extreme Feedforward Learning machines	Blockchain and FL-based model	[24]
	2960 CXR and 746 CT/GhostNet, ResNet50, ResNet101	Reduce communications and improve the model’s performance	[21]
	COVIDX8/ResNet18	FedFocus framework for improving the training efficiency	[20]
	108 CXR/VGG16 and ResNet50	COVID-19 detection with Non-IID and unbalanced data distributions	[9]
	DarkCOVID and ChestCOVOD/GAN	COVID-19 detection with joint design of GAN and FL	[6]
	COVID19ACTION-RADIOLOGY-CXR dataset/ResNet50/DenseNet121/InceptionV3/Xception	IoMT for ease of access	[25]
	COVID-FL dataset/Transformer model	Self-supervised learning for data heterogeneity	[4]
	Multiple CT datasets/RetinaNet	Model generalizability on unseen data	[22]
	34,006 CT images/Capsule network	Blockchain based FL	[23]
Pneumonia	Pediatric pneumonia dataset/ResNet18	Privacy preserving deep learning on multi-institutional X-ray images for multiclass classification	[27]
	Public dataset/Custom CNN model	Scalability of intermittent clients with pneumonia classification CXR images	[28]
Tuberculosis	Chest X-ray14, Tuberculosis Chest X-ray, COVID-19 detection dataset/DenseNet121	Federated partially supervised learning foe clients with limited labeled data	[29]

#### 3.1.2. Cancer

This section describes the use of FL techniques for cancer diagnosis, with a summary provided in Table 2. A high breast density is indicative of around five times increased risk of breast cancer [17]. FL with the FedAvg algorithm [12] was used for breast density classification and showed improvement in results with FL models of 6.3% on average above the models trained only on the local data [17]. DenseNet-121 model was used with a classifier for the four BI-RAD categories [17]. Breast histopathology image (BHI) dataset was used for Invasive Carcinoma of No Special Type (IC-NST) detection using ResNet model [30]. The model performance improved by combining the Gabor and ResNet features, and provided similar performance to the other selected studies [30]. The model’s generalization was demonstrated using the breast cancer histopathological (BreakHis) dataset [30]. BreakHis dataset was also used for breast cancer classification and found the FL results to be comparable to centralized learning [31]. The participating clients may have different domain data which can be used in FL to solve various tasks [1]. The proposed multi-domain and multi-task FL approach was evaluated for tumor segmentation on breast mpMRI dataset, and achieved an overlap of 0.65 for lesion segmentation [1].

Six pre-trained models were used for brain tumor classification on MRI images [32]. Three out of six (DenseNet121, VGG19, and Inception V3) models were selected as an ensemble, and provided better results compared to the selected studies. Although the FL provided a slightly lower performance compared to the average CNN model, but was privacy-preserving [32].

A Message Queuing Telemetry Transport (MQTT) based networking framework was proposed for FL [33]. MQTT protocol was used for the exchange of the U-Net model parameters. The proposed methodology was tested for brain tumor segmentation on BraTS dataset using U-Net [33]. The proposed system used asynchronous consensus and benefitted using the scalability, bandwidth efficiency, and reliability of the MQTT protocol [33]. A FL model, FedGIMP, was proposed for multi-site collaborations with decentralized learning of generative MRI priors [34]. The proposed method was used for MRI reconstruction and was compared against other models on multiple datasets, achieving better performance for Peak Signal-to-Noise Ratio (PSNR) and Structural Similarity (SSIM) [34]. A FL method SplitAVG was proposed to address the data heterogeneity issues for brain tumor segmentation on BraTS 2017 dataset [35]. The proposed optimization platform was compared against seven state-of-the-art FL methods, demonstrating the effectiveness of SplitAVG by requiring simplified hyperparameter tuning and lower requirement of additional training [35]. The FL approach was used for brain tissue classification on BraTS 2017 dataset with data from ten institutions, achieving a model accuracy comparable to the centralized approach [3].

A Generative Adversarial Network (GAN) was proposed for stain-style normalization for multiple clients for histopathology images of colorectal cancer (CRC) [36]. The proposed method was comparable in comparison to a centralized model, and provided a 20% accuracy increase over the baseline classification model [36].

Histopathological images from The Cancer Genome Atlas (TCGA) dataset were used to investigate differential private FL for IID and non-IID distributions [11]. The proposed method used differential privacy and multiple instance learning (MIL). It was demonstrated that differential privacy can improve the performance of the image analysis [11]. Similarly, whole-slide image classification of histopathological images was used with a multiple instance learning (MIL) at a local client [37,38]. The study used hyper-network in the central server to learn the model from the client’s networks, with noise added to the raw data from the clients [37]. The study investigated prostate cancer dataset PANDA, and TCGA-NSCLC and LUSC lung dataset for cancer classification with FedAvg as a baseline method for comparison [37]. The hyper-network achieved an accuracy of 0.957 and 0.920 for the prostate and lung cancer, respectively [37], which was comparable to centralized model [37]. A Customized FL (CusFL) was proposed for a decentralized prostate cancer classification on PROSTATEx and LocalPCa datasets with a custom CNN model [39]. The proposed CusFL method provided better accuracy compared to other selected FL methods, such as MOON and SplitNN, with different number of participating clients [39]. A Variation-Aware FL (VAFL) was proposed to address the inter-client variations of the image data [40]. The client with the lowest data complexity was chosen first and the images were transformed to a common image space to synthesize images using a Generative Adversarial Network (GAN) [40]. The proposed framework was used for prostate cancer classification and the results for VAFL were found better than centralized and local learning [40].

A blockchain-based decentralized FL framework was proposed for lung cancer classification with EfficientNetB7 on LC25000 lung and colon cancer histopathological dataset, achieving an accuracy improvement over decentralized model [41]. Network Architecture Search (NAS) was proposed to be used in combination with FL for medical data security [42]. NAS is a technique to find the best architecture with the optimum parameters. A multi-objective fuzzy FL model (CIT2FR-FL-NAS) was proposed and the model was tested on LC25000 for lung and colon histopathological image dataset and showed high accuracy compared to the other considered models [42].

A study investigated thyroid cancer detection on 8457 ultrasound images from six healthcare institutions [43]. The study used five deep learning models and performed external validation on images from another dataset [43]. The results showed that the FL model performed comparably to the centralized learning [43].

**Table 2:** FL applications for cancer.

Organ	Dataset/Model	Study Focus	Ref
Breast	BI-RADS/DenseNet-121	Breast density classification into four classes	[17]
	BreakHis/ResNet-152, DenseNet-201, MobileNet-v2-100, EfficientNet-b7	Breast cancer histopathological image classification	[31]
	Breast mpMRI, Brain mpMRI/U-Net	Multi-domain model for lesion segmentation	[1]
	BHI dataset/GaborNet and ResNet	Classification and model generalization	[30]
Brain tumor	UK data service (MRI) CNN Ensemble of VGG16, InceptionV3, VGG19, ResNet50, Xception, and DenseNet121	Brain tumor classification with federated and centralized learning	[32]
	private dataset and IXI, fastMRI, BraTS/unconditional adversarial model with eight fully-connected layers	MRI reconstruction with decentralized training of generative image priors	[34]
	BraTS2017/U-Net model	Distinguishing healthy and cancerous brain tissues	[3]
	BraTS dataset/ResNet34	Model performance drop issues with data heterogeneity	[35]
	BraTS 2018, BraTS 2020 datasets, ATHENS dataset (private)/U-Net	Networking framework for Brain tumor segmentation with MQTT protocol	[33]
Prostate	Prostate cancer dataset PANDA, and TCGA-NSCLC, LUSC lung cancer/	Prostrate and lung cancer detection with hyper-MIL network	[37]
	Prostate cancer dataset/Custom CNN model	Handling inter-client variations	[39]
	LocalPCa and PROSTATEx challenge dataset	Handling inter-client variations for Clinically significant prostate cancer classification	[40]
Lung	LC25000 Lung and colon cancer histopathological images/EfficientNetB7	Privacy preservation with blockchain for lung cancer detection	[41]
	LC25000 lung and colon histopathological image dataset/CIT2FRNN model	Use of Network Architecture Search (NAS) with FL for model architecture selection and privacy protection	[42]
	TCGA Dataset/Attention based MIL, DenseNet	Effect of IID and non-IID distributions	[11]
Kidney	Renal cell carcinoma (RCC), Breast Invasive carcinoma ((BRCA)/Multiple instance learning	Weakly supervised attention multiple instance learning FL for whole slide images	[38]
Thyroid	Thyroid ultrasound images from 6 institutions/VGG19, ResNet50, ResNext50, SE-ResNet50, SE-ResNext50	Comparison of FL to centralized learning for real-world healthcare	[43]
Colorectal	Cancer Genome Atlas (TCGA), CRC-VAL-HE-7K, NCT-CRC-HE-100K	multiple-client based stain-style normalization	[36]

#### 3.1.3. Skin

A skin lesion classification used the HAM10000 dataset and achieved 76.9%, which was similar in classification accuracy to the selected schemes, but provided better privacy with homomorphic encryption, and could better handle the client dropout [44]. The proposed scheme was evaluated against four other aggregation and encryption schemes [44].

A customized FL (CusFL) model was proposed with an objective to handle inter-client variations with a single federated model [39]. The proposed model was compared with other techniques such as SplitNN and achieved better performance due to the guiding of the private model training with federated feature extractor, and feature alignment with the global model [39].

A multiclass classification of the skin diseases was performed on images from the DermNet dataset and the proposed FL approach achieved better performance with an accuracy of 94.15% with 2500 clients [45]. The results showed an improvement in the FL performance with an increase in the number of clients [45]. MRI dataset, HAM10000 was used to overcome the challenge of the performance degradation with FL [46]. This was addressed by a server-side Progressive Fourier Aggregation (PFA) for gradual aggregation of the model parameters in the frequency domain, and reducing the local class imbalance based on the global imbalance [46]. The results showed better performance compared to the other selected FL models, such as, FedAvg, SiloBN, and FedProx [46]. A fuzzy consensus-based framework was proposed for the skin disease classification on the HAM10000 dataset [47]. Many classifiers were used for making the decision, and the results showed an accuracy of 89.12% and that using many classifiers improved the results by 0.5% compared to a single classifier [47].

The performance degradation due to data heterogeneity, and lack of labelled data in FL was addressed by a Transformer-based self-supervised model on dermatology ISIC-2017 dataset and achieved 1.53% improvement compared to supervised baseline [4].

#### 3.1.4. Eye

The performance of FL framework was evaluated for the segmentation and classification of Diabetic Retinopathy with DNN using Optical coherence tomography (OCT) and OCT Angiography Data. The results were found to be comparable to centralized learning [48].

The data distributions across institutions are heterogeneous and can affect FL performance [35]. A heterogeneity-aware method was proposed for diabetic retinopathy that achieved comparable performance to the centralized models, and was found better compared to the other selected FL models [35].

A transformer based self-supervised framework was proposed for diabetic retinopathy detection [4]. It was shown that the proposed model was better at generalizability and handling limited labelled data [4].

For a summary, please see Table 3.

#### 3.1.5. Heart

MRI images were analyzed using 3D-CNN for hypertrophic cardiomyopathy diagnostics [49]. It was shown that with a small dataset for cardiac MRI comparable performance to centralized learning can be achieved using FL [49]. The segmentation masks were provided by the clinicians [49].

The problem of having limited labeled data was addressed using contrastive learning to learn from the unlabeled data [50]. The MRI images MICCAI 2017 challenge dataset was used for image segmentation with U-Net model [50]. The segmentation and labelling results were significantly better than the selected state-of-the-art techniques [50].

For a summary, please see Table 3.

#### 3.1.6. Brain Disorders

A Gradient Matching Federated Domain Adaptation (GM-FedDA) framework was proposed for fMRI image classification using SCZ and DMM datasets for determining brain disorders [52]. The proposed model outperformed the selected methods including Principal Component Analysis (PCA)/Support Vector Machine (SVM), local, and FedAvg [52].

The Autism Brain Imaging Data Exchange (ABIDE) dataset was used for Autism Spectrum Disorders (ASD) classification with privacy-preserving FL implemented with a randomization mechanism for sharing the local model weights [10]. A Federated Multi-Task Learning (MTL) framework was proposed for the diagnosis of multiple mental disorders on MRI data from ABIDE, ADHD-200, COBRE datasets [51]. The study used MLP with contrastive learning and demonstrated reliability and effectiveness with limited computation resources [51].

For a summary, please see Table 3.

### 3.2. Overcoming Security Threats

The chances of an attack by malicious agents are lower in the case of in-silo FL, with major healthcare institutions collaborating to develop a global model, as the membership would be restricted, with the identity and contributions of the participants known. This, however, may be difficult to enforce for on-device collaborations where any device can contribute data with the possibility of malicious intent.

It is often assumed that with FL, sending the gradients and not the data to a central server can preserve privacy; however, it is possible to recover the images from the gradients [53,54]. The data privacy techniques of homomorphic encryption and differential privacy are aimed at protecting the sensitive data for privacy preservation [41]. The details of the privacy mechanisms and how to measure their effectiveness is covered in [26,44,55]. In this section, we describe the attacks and defense against them, with a summary provided in Table 4.

#### 3.2.1. Poisoning and Inversion Attacks

The participants in the FL process requires regular communications between the clients and the server, and is susceptible to malicious parties that can alter the learning process [56]. In poisoning attack, the adversary can corrupt the model updates to the server, or alter the client training datasets [57]. A Distance-based Outlier Suppression (DOS) algorithm was proposed for protection against different untargeted poisoning attacks on FL [56]. ResNet18 model was used with ten FL clients on CXR and dermoscopic images showed that the proposed method had better performance with up to 50% clients experiencing byzantine failures [56].

A gradient inversion attack tries to match the trainable input data and the real data [27,58]. The case of multi-site fMRI brain data classification was studied with a view to enhance privacy using a randomization mechanism to modify the model weights [10]. Gaussian and Laplace randomization noise level determined the privacy level, and it was determined that the model failed in the classification task for higher noise level corresponding to higher privacy preservation [10]. It is important to quantify the possibility of such an attack, and an improved Rank Analysis Index (RA-I) was proposed for this purpose [54]. A study investigated the nature of the threat by gradient inversion attacks to FL and provided insights to the trade-offs between the model’s accuracy and privacy-preserving techniques, such as differential privacy [58].

A CXR image classification task with a pre-trained ResNet-18 model was used to investigate server-side model inversion attacks, considering the batch normalization (BN) updates, usually not considered in similar studies [58]. The study showed using a simple mechanism of adding Gaussian noise to the model updates can protect against the inversion attack; however, it also reduces the model’s accuracy in the process [58]. The sharing of local model weights modified using a randomization mechanism and an iterative optimization algorithm were proposed to safeguard against the gradient attacks on fMRI data [45].

A secure framework MediSecFed was proposed for secure medical image classification in hostile environments [59]. The performance of the proposed MediSecFed with FedAvg algorithm showed an improvement of 15% on two selected chest X-rays datasets in the presence of malicious clients [59].

#### 3.2.2. Homomorphic Encryption

Homomorphic Encryption is used to apply certain mathematical operations directly to the encrypted data. This is helpful for keeping the values hidden during sharing by the participants [57].

Although homomorphic encryption provides a privacy preservation guarantee, it also has an associated computational overhead that can have a significant impact for a complex model. Thus, using homomorphic encryption can be difficult for such cases [44]. A privacy-preserving scheme was proposed based on masks and homomorphic encryption and the results were evaluated on the skin lesion dataset to overcome the limitations of homomorphic encryption [44].

#### 3.2.3. Differential Privacy

Differential privacy is a standard approach to mitigate privacy risks and is achieved by adding noise or perturbations to the local data, but these local privacy approaches can often come at a cost to the accuracy [41,57]. Differential privacy can prevent the leakage of personal information by introducing uncertainty in the model [26].

A framework was proposed to address the privacy issues of sharing histopathological images with differential privacy [11]. The study used Cancer Genome Atlas (TCGA) dataset distributed across seven clients, and demonstrated that the distributed training can achieve similar performance to conventional centralized training, and yet provide the data privacy safeguards [11].

In a study for COVID-19 detection, differential privacy was used by each participating hospital to enhance the privacy of the COVID-19 data [6]. The evaluation of the model’s accuracy found it to be slightly lower with the use of differential privacy, and the amount of noise added was found to determine the quality of FL training [6].

#### 3.2.4. Multi-Party Computation (MPC)

MPC allows the participating clients to compute the aggregated model without a central server, which can provide better model parameter privacy [16]. MPC is implemented in the PySyft library and although it prevents the model leakages, the aggregation center can still recover the model or recover the local training images [53]. An Augmented Multi-Party Computation (AMPC) method was proposed for secure FL systems [53]. Although standard MPC can provide protection such as differential privacy, it can fail in certain scenarios [53]. The proposed method provides for two decomposition rounds for encrypting the local models before communicating these to the server [53]. The authors demonstrated the model’s efficacy with theoretical analysis and empirically using the MNIST and CIFAR-10 datasets [53].

The proposed framework, PriMIA used MPC for remote inference demonstrating the protection of data and model privacy for pediatric pneumonia classification [27].

**Table 4:** Approaches for the FL security.

Defense for	Dataset	Study focus	Ref
Poisoning attacks	CheXpert and HAM10000	Distance-based Outlier Suppression (DOS)	[56]
Model reconstruction/inversion attacks	CXR dataset/ResNet18	Gradient inversion attacks on FL use cases	[58]
	COVIDX-8a and COVIDX-8B/ResNet18 and ResNet34	Privacy preserving with proposed MediSecFed in presence of malicious clients	[59]
	Pediatric pneumonia classification/ResNet18	End-to-end privacy preserving FL	[27]
	FMRI ABIDE dataset	Privacy preservation with randomization and optimization algorithm	[10]
Differential privacy	TCGA Dataset/Attention based MIL	Differential privacy on histopathological images with seven clients	[11]
	COVID-19 detection	Differential privacy at each hospital	[6]
	COVID-19 detection/custom CXR dataset	Differential privacy stochastic gradient descent (DP-SGD) for data privacy	[26]
Homomorphic	HAM10000	Homomorphic encryption, masks for local model protection	[44]
MPC	MNIST and CIFAR-10	Augmented MPC with encryption	[53]
	Pediatric Pneumonia classification	Data and model privacy	[27]

### 3.3. Improvement Strategies

#### 3.3.1. Performance Improvement

The performance and scalability of pneumonia classification with CXR images was studied for the case of intermittent clients, that is, variations in the number of clients [28]. The clients may join to participate in the training cycle or they may drop out. The proposed approaches that handle the data of such clients showed improvements in accuracy compared to a centralized approach along with a reduced computing time; however, the communications cost was not considered in the study [28]. A customized FL (CusFL) was proposed with each client learning a customized model by leveraging the collective federated model [39].

The proposed Federated Multi Task Learning Framework for Joint Diagnosis (FMTLJD) was used for multiple mental disorders diagnosis, showing that the shared knowledge of the mental disorders can improve generalizability [51]. The effect of adding the clients incrementally was also investigated, with a total of eight participating clients with different sample sizes [51]. It was shown that the proposed method worked for the institutions with small datasets for effective learning [51].

#### 3.3.2. Addressing Labelling Issues

Semi- or weakly supervised learning can be used for model training with some unlabeled data. The lack of labeling in the datasets were addressed using a self-supervised learning method [4]. A semi-supervised technique was implemented for COVID-19 detection using a U-Net-based encoder–decoder architecture [14]. A weakly supervised classification using multiple instances learning framework was proposed for gigapixel whole slide images for renal and breast carcinoma detection [38]. The results showed that for the unseen data, the FL model generalized better compared to models from a single institution [38].

A method named FedCy was proposed for surgical phase recognition on a multicenter cholecystectomy video dataset from 2022 in federated semi-supervised learning using ResNet-50 [18]. The performance of the proposed method was better on the unlabeled datasets than the state-of-the art method FedRGD [18].

Partially Supervised Learning (PSL) is useful with the clients that have partially labeled data [29]. A framework FedPSL was proposed to overcome the challenges with Federated Partially Supervised Learning (FPSL) to overcome the problem where the clients in FL have only limited partially labeled data [29]. The proposed method demonstrated robust performance compared to the baseline methods under data scarcity and domain shifts challenges [29].

#### 3.3.3. Model-Contrastive FL

The FL requires labelled data for training the global model which can be a big constraint. Contrastive Learning (CL) approaches can be used to learn from unlabeled data considering the availability of the limited labelling for fine-tuning [50]. A federated contrastive learning (FDL) approach was proposed to overcome the limitation of labelled data with participating clients in volumetric image segmentation [50].

A model-contrastive FL framework, MOON, was proposed to exploit the models’ similarities to improve local training of models, and to address the data heterogeneity [60]. The framework used contrastive learning for unsupervised training on the unlabeled videos and was shown to perform better in comparison to other FL algorithms on the selected datasets [60].

A framework named FMTLJD, based on multi-task contrastive learning (MTL), was proposed for multiple brain disorders with a contrastive feature extractor for extracting high-level features across the models [51]. The proposed framework was compared with the selected models such as Fed-MoE, and Fed-Align, and was shown to address the domain shift between clients [51].

#### 3.3.4. Incremental Learning

The application of deep learning can be difficult for cases with a continuous medical data stream and requires time and space [61]. Incremental Learning (IL) is a variation of FL where the model is trained with data from one institution, and then is successively trained with the next participating institution [3]. One disadvantage of this method is that the patterns learnt from the previous institutions’ data can be disregarded once the model is trained with data from the next institution [43].

The use of FL in conjunction with incremental learning was proposed, with incremental learning able to process a stream of new data without forgetting the old knowledge learned earlier [61]. FL was combined with exemplars from incremental learning and shown to reduce the time and space [61].

## 4. Open Research Challenges

This section describes the open research challenges relating to the application of FL techniques for medical imaging.

### 4.1. Communications

Different participating units may have different processing and network bandwidths which can affect the timely model updates and the client may not be able to participate in all the training rounds.

The differences in computational power of the participating devices can be a hindrance in the FL applications. To overcome this challenge, PruneFL was proposed for adapting the model size using an adaptive parameter pruning. The approach was shown to reduce the communication and computation overhead using Raspberry Pi-4 as edge devices for the various datasets, with similar model accuracy [62]. A dynamic approach was proposed to overcome the communications challenge with the clients selected for model updates based on their local model performance [21]. Thus, a local client only sends updates if the local model performance has improved [21]. The communications cost was reduced by proposing a model FCLOpt, which was not reliant on negative samples, for an optimized federated contrastive learning [50]. The model was used to reduce model downloading communications with the proposed predictive target network update (PTNU) [50].

The proposed method, MediSecFed used logits instead of model parameters, thereby making the communications more efficient especially for low bandwidth [59]. The communications overhead was reduced using the proposed FetchSGD model by improving the communications bottleneck [63]. Sketching allowed the sending of momentum and error from the client to the central server. The study provided theoretical foundations and also empirically evaluated the proposed scheme using ResNet9 and ResNet101 for CIFAR10/100 and FEMNIST datasets and showed better performance compared to the selected baselines [63].

### 4.2. Data Heterogeneity

The data from different institutions can be heterogeneous. The data available at the participating clients should be IID for the global model training, however this assumption might not be correct [60,63]. The data for FL is generally not IID, because the data distribution of the different participating clients differs due to various reasons, such as location, local population, etc. The performance drop due to data heterogeneity across the participating institutions was addressed with SplitAVG, a heterogeneity-aware FL method [35]. The proposed method was compared with seven other FL methods, such as FedAvg, and splitNN, for diabetic retinopathy, bone age, and brain tumor segmentation tasks, and showed better performance in heterogenous data handling [35].

The effect of IID and non-IID distributions on FL performance was studied using the TCGA dataset [11]. The training image data for this investigation was created by randomly dividing the images between the different clients [11]. The efficacy of FL with FedAvg was demonstrated for both IID and non-IID data distributions [11]. The non-IID issue was studied by proposing a model-contrastive learning framework [60]. The non-IID data was addressed through an optimization-based method and the performance was evaluated on the multiple datasets [4]. Domain shift can be a challenging issue when the various participating institutions have heterogenous data distributions [10].

### 4.3. Data Bias

Unbalanced sets can be a challenge for training the FL models and can degrade performance [46,64]. Biases in the data can be introduced due to the underrepresentation of some strata of the patients. This can affect the training of the DNN models which then learn these biases. The data bias in the data from the local clients can disrupt the global model. The bias was reduced by partitioning the dataset of each client into five folds and ensuring that the 2D scans of a patient are included in each fold [3].

A solution was proposed to address the class imbalance using Conjoint Prototype Aligned (CPA) loss for a balanced optimization of the FL framework on MRI datasets [46]. CPA loss was used to adjust the client-side local training by determining the global conjoint objective from the global imbalance [46]. The results showed an improved performance compared to the other selected approaches [46].

### 4.4. Blockchain

Blockchain provides tamper proofing and immutability of the data by maintaining the data in a decentralized manner; thus, it can guard against the central server failure. Similar to FL, blockchain is a decentralized technology and can be used with FL to improve the privacy and security of the data.

Blockchain FL was proposed for data authentication in the model sharing [23]. A blockchain privacy preserving framework was proposed with the model’s parameters were shared on the blockchain using smart contracts, and the models were stored off-chain [41]. A blockchain-based framework FedGAN was proposed for secure COVID-19 detection [6]. Each edge node setup a wallet for public and private keys, and delegated proof-of-stake (DPoS) was used as a consensus mechanism [6].

### 4.5. Institutional Differences

In medical imaging, standards such as Digital Imaging and Communication in Medicine (DICOM) and Picture Archiving and Communication System (PACS) for data storage and archival are used; however, the other related procedures and equipment at the various participating institutions might be different. The processes for image acquisition, labelling protocols, and even the hardware used may have differences amongst the various participating healthcare institutions. This can have an effect on the performance of the FL models.

The stain-variation issue for the histopathological images can be an issue as different institutions use different stain styles [36].

## 5. Conclusions

Although the federated learning research area is still growing, the benefits of creating better generalized global models for the healthcare domain will result in facilitating better disease diagnosis and saving precious lives. The global models can be standardized, making these optimal diagnostic models widely accessible to healthcare establishments irrespective of their size, location, and contribution in model training. With novel and improved schemes for protecting gradients communications to the global server, secure and private data sharing across the communications channels will become possible.

The aggregated model post training at the central server and in deployment at the local server has the potential to be re-trained, as more data becomes available locally, and in cases of model drift.

## Figures and Tables

**Table 3:** FL applications for various diseases.

Disease/Organ	Dataset/Model	Study Focus	Ref
Eye	Multiple datasets/residual U-Net and VGG19	Diabetic Retinopathy Classification of OCT and OCT Angiography Data with small datasets	[48]
	Diabetic Retinopathy dataset/	Model performance drop issues with data heterogeneity	[35]
	Kaggle Diabetic Retinopathy dataset/Transformer model	Self-supervised learning for data heterogeneity	[4]
Prostate	Prostate MRI dataset from NCI-ISBI 2013 and PROMISE12/VGG-16BN and ResNet-18	Image segmentation with class imbalances addressed by handling data heterogeneity, achieving a 4% increase in Dice score	[46]
Kidney	WB PET-CT images/U-Net	Kidney localization with multi modal data (PET, CT) demonstrating feasibility of training a general AI framework for the unique domain and tasks by the clients	[1]
Heart	MRI M&M and ACDC dataset subsets/3D CNN ResNet-18	Diagnosis of hypertrophic cardiomyopathy	[49]
	ACDC MICCAI 2017 and HVSMR MICCAI 2016/U-Net	3D cardiac image segmentation	[50]
Skin	HAM10000/DNN	Skin Lesion Classification	[44]
	HAM10000 dataset/ResNet18	Skin lesion classification for handling inter-client variations	[39]
	DermNet dataset with four classes, 849 images/VGG16 and AlexNet	Classification of four diseases: acne, psoriasis, eczema, and rosacea	[45]
	Dataset based on HAM10K and MSK/ResNet-18	Personalized framework for addressing performance degradation and class imbalance	[46]
	HAM10000/VGG and Inception	Skin classification with fuzzy consensus multi classifiers	[47]
	ISIC17 dataset/Transformer model	Self-supervised learning for data heterogeneity	[4]
Brain disorders	ABIDE, ADHD-200, COBRE/Expert network as a neural network stack	Joint diagnosis of mental disorders with contrastive learning	[51]
	SCZ and MDD dataset/Adversarial domain adaptation	Gradient matching-based Neuropsychiatric disorder classification	[52]
	ABIDE/Multi-Layer Perceptron	Privacy preserving fMRI analysis for identifying Autism Spectrum Disorders (ASD) or Healthy Control (HC)	[10]

## Data Availability

No new data were created or analyzed in this study. Data sharing is not applicable to this article.
